# Neuropathology of HAND With Suppressive Antiretroviral Therapy: Encephalitis and Neurodegeneration Reconsidered

**DOI:** 10.1007/s11904-015-0266-8

**Published:** 2015-04-10

**Authors:** Benjamin B. Gelman

**Affiliations:** Departments of Pathology and Neuroscience & Cell Biology, University of Texas Medical Branch, 301 University Blvd., Keiller 3.118A, Route 0609, Galveston, TX 77555-0609 USA

**Keywords:** Antiretroviral therapy, Dementia, Encephalitis, HIV/AIDS, HIV-associated neurocognitive disorders, Metabolic encephalopathy, Neurodegeneration, Neurovirology

## Abstract

HIV-1 infiltrates the central nervous system (CNS) during the initial infection and thereafter plays a persistent role in producing CNS dysfunction as the disease progresses. HIV-associated neurocognitive disorders (HAND) are highly prevalent in HIV-infected patient populations, including currently infected patients with good access to suppressive antiretroviral therapy (cART). cART decreased the severity of CNS dysfunction dramatically and, in doing so, upended the neuropathological foundation of HAND pathophysiology. It is clear that the working concept of pathophysiology prior to cART, which was driven by inflammation, encephalitis, and neurodegeneration, needs to be replaced. The NeuroAIDS field is reluctant to take that important step. This review explores the fact that the neuropathological concept that drove the field before the era of cART no longer seems to fit with what is commonly observed in patients treated successfully with cART. The field clings to the pre-cART idea that HAND is sequentially driven by virus replication in CNS, brain inflammation (encephalitis), and neurodegeneration. Neurovirological, clinicopathological, and gene expression correlations in cART-treated patients, however, provide little strong support for it. Introducing cART into clinical practice decreased HIVE, inflammation, and degeneration but did not cure HAND. Brain gene array data suggest that the neurovascular unit is a critical target in virally suppressed patients with HAND. The NeuroAIDS field needs an infusion of new ideas to steer research toward issues of the highest relevance to virally suppressed patients. With no suitable replacement immediately within reach, devaluating formative ideas is understandably difficult to accept. The cliniconeuropathological correlation in virally suppressed patients needs to be better defined.

## Introduction

Central nervous system (CNS) infection is established early in the course of HIV/AIDS. CNS pathology was a highly prevalent feature in the natural history of patients who were not virally suppressed. The topic of HIV infection of the CNS has enjoyed a sustained period of scientific and clinical interest for over 30 years. The neuropathology of end-stage AIDS has been illustrated comprehensively and is well-described in many excellent book chapters and review articles [1, 2]. Images of the histopathology can be easily acquired in cyberspace in these times and there seems little need to illustrate them yet again. Updating a fast-moving field is potentially useful nevertheless to discuss the existing body of knowledge with a changed perspective. Current times have not been propitious for the discovery of exciting new developments in the neuropathology of HIV/AIDS. The paucity of recent articles with high impact makes an update of neuropathology challenging, but also heightens opportunities for analysis, reflection, and commentary. The need for new and genuinely novel ideas in this field has never been greater. HIV-infected patients and the new corps of enterprising young practitioners both deserve to inherit a scientific legacy that points in a clear direction that focuses on relevant problems. Accordingly, this article provides commentary with the aim of spurring new investigators to propel the field creatively forward.

There is a virtual haze of comorbid changes and/or age-related pathologies that can occur in HIV-infected subjects. When one wipes away the haze in patients who are compliant with taking suppressive antiretroviral therapy (cART), the overwhelmingly dominant HAND-associated neuropathology observed at autopsy is “no specific neuropathology.” The lack of a specific neuropathological change, which became the most common outcome at autopsy at the close of the twentieth century, has led to a dearth of novel breakthroughs in the neuropathological literature for at least 15 years. The medical literature favors positive data and shuns negativity. Composing a scholarly treatise regarding the disappearance of pathologies that were prevalent formerly is perhaps more challenging than describing the panoply of CNS changes observed before the era of cART. Present circumstances in the era of cART have benefited our patients substantially, but we still have not canonized a new cliniconeuropathological correlation for HIV-associated neurocognitive disorders (HAND). With no clear-cut replacement for the “old” neuropathology to guide us in the era of cART, one can reasonably wonder what the actual mechanism of HAND really is. Therapeutic strategies of current interest are based primarily upon neuropathologies that do not occur with high prevalence. Given the low prevalence of those pathologies in virally suppressed patients, their clinical relevancy to HAND, which has an estimated prevalence of up to 50 % [3], becomes increasingly dubious.

From a historical perspective, the most important cliniconeuropathological concept in the field was established prior to the era of cART in patients in whom virus replication was not suppressed medically. In those patients, progression to end-stage AIDS was virtually certain to occur and about 20 % of patients had HIV encephalitis (HIVE) at autopsy [4, 5]. This neuropathological lesion in the brain was first labeled a “subacute” type of inflammatory process characterized by several features in combination, the most important of which was evidence of HIV replication in mononuclear phagocytes (macrophages and microglial cells).

## Microglial Nodules

Foremost among the histological changes in HIVE was the presence of microglial nodules (MGN), which are activated microglial cells arranged in clusters of variable sizes. Microglial cells are the resident histiocytes of the CNS and, combined with brain macrophages, are the major reservoir of replicating HIV in the brain. Unlike macrophages which originate in bone marrow, microglial cells arrive early in development from the yolk sac and can reproduce locally in the CNS [6]. MGNs can occur in either gray or white matter almost anywhere in the CNS. They can be striking in their abundance in the tissue but are not diagnostic for HIVE because they may be observed in several other kinds of brain infection. When the MGNs are immunostained for HIV proteins such as gp41, HIVp24, or gp120 they are considered to be specific to HIV infection of these cells. Over the years, some confusion was produced because case material often contained an abundance of MGNs without other lesions associated with HIVE (to be discussed). These cases still may be diagnosed “microglial nodule encephalitis” (MGNE) in lieu of HIVE because other infectious agents could not be readily ruled out. It was a critical diagnostic dilemma prior the cART era because encephalitis caused by opportunistic pathogens such as cytomegalovirus and *Toxoplasma ghondii* also produced MGNs in immunosuppressed patients with AIDS [1, 2, 7••]. The neurovirological underpinnings of HIVE and MGNE diagnoses were recently unraveled. It was shown that HAND with HIVE and HAND with MGNE both are associated with high rates of HIV replication (HIV RNA) in the brain. As expected, brain HIV replication is much higher in HIVE than MGNE, which suggests that there is a neuropathological progression of the disease from lower to higher replication rates. In patients with HAND without either of the two diagnoses, brain HIV RNA and DNA were not significantly higher than patients without HAND. Interestingly, HAND without these two pathologies was significantly correlated with HIV replication in blood plasma, but not in the brain or CSF. Apparently, systemic viral suppression is just as or more critical than viral suppression within the CNS in these patients [7••].

## Multinucleated Giant Cells and Macrophages in HIVE

The “hallmark” lesion of HIVE is the multinucleated giant cell (MNGC) [5]. These cells represent mononuclear phagocytes productively infected with HIV that have undergone cellular fusion mediated by the HIV fusion protein gp41. MNGCs are most often observed around blood vessels and resemble other kinds of macrophage giant cells in their general appearance such as those seen in granulomatous inflammation. They are readily distinguishable from other kinds of macrophage giant cells by their positive immunostaining with antibody against HIV proteins, the lack of associated granulomatous inflammation, and the clinical and pathological context in which they are observed. This feature alone is generally adequate to make the diagnosis of HIVE in an infected patient; other features of HIVE described herein are almost always present as well. Many single macrophages and/or clusters of macrophages are usually present in HIVE. They are most often seen around the blood vessels in white matter and often contain hemosiderin pigment. Macrophages productively infected with HIV-1 in the brain generally have an M2 (“repair”) phenotype; they are CD163+, CD14+/CD16+, and they contain stainable HIV-1 antigens [8, 9].

## Other Inflammatory Cell Changes in HIVE

Ancillary and often nonspecific neuropathological changes beyond the key features mentioned above can contribute to a wide spectrum of changes in HIVE. In isolation, these changes are seldom specific enough to diagnose HIVE, but when observed in combination with other features they contribute to a highly characteristic constellation of changes. Diffuse microglial cell activation (“diffuse microgliosis”) is commonly observed in HIV-infected brain tissue. It refers to an increase in the number of cells with a dark-stained rod-shaped nucleus scattered in the brain tissue. An increase in the density of “rod cells,” which are the elongated hematoxylin-stained nuclei, can occur with or without the formation of MGNs. They are usually present in HIVE and MGNE, but frequently they are observed without either of the two diagnoses. The expansion and heightened ramification of the cytoplasmic processes of the activated microglial cells are not visible in routinely stained tissue sections but are recognized with immunostaining for HLA-D, Iba-1, ferritin, and other antigens [10, 11]. Other inflammatory cells that can be observed in HIVE include scattered perineuronal lymphocytes. CNS T lymphocytes are not required to make the diagnosis of HIVE and they lack specificity when observed. Indeed, their presence in high abundance serves to heighten suspicion that another agent that can cause encephalitis might be present. Even though lymphocytic infiltration is seldom the most telling or prominent, brain lymphocyte subsets conduct regular immune surveillance in the CNS and still could play a seminal role in the pathophysiology of HIVE such as with neuronal killing [12]. Infiltration of the brain with acute inflammatory cells such as neutrophils and eosinophils in HIVE is not likely to be important because they do not host HIV infection. The basic nature of the inflammatory reaction to HIV infection in the CNS is generally considered to be “subacute” given the lack of neutrophils.

## Astrocyte Changes in HIVE

In HIVE astrocyte hypertrophy and glial scarring are observed best in brain white matter. The hypertrophy is a nonspecific reaction that occurs in all types of brain injury. HIV-infected astrocytes are not morphologically different in a routine neuropathological examination. Diagnostically, their chief value is in establishing that damage has occurred in the setting of inflamed brain tissue. The role of astrocytes remains of great interest because in vivo and in vitro data indicate that astrocytes can be infected with HIV [13]. They also can participate in antigen presentation [14]. HIV-infected astrocytes do not produce assembled viruses very effectively and are not likely to be the main contributor to CNS HIV replication rates. Astrocyte infection is very important because of its potential ability to support viral latency in the brain. In cART-treated and naïve patients both, reverse transcribed HIV DNA is integrated into the host cell genome and becomes “latent” in a highly limited number of host cells. Virally latency is defined as HIV DNA integrated into the host cell genome that is transcriptionally silenced and immunologically inert, yet capable of transcriptional reactivation to produce assembled viruses that are fit to infect other host cells. Upon stopping cART, it is the latent pool of HIV DNA that “reseeds” active HIV replication and prevents the complete eradication of HIV infection. Data thus far indicate that integrated HIV DNA is present in a substantial number of astrocytes in patients with HIVE [15••]; it remains unclear whether the astrocyte pool of HIV DNA contributes to the pool of latent HIV that reseeds the CNS with replicating HIV when cART is stopped. If so, the astrocyte reservoir of latent HIV is a highly significant pool of virus that would need to be dealt with in therapies to eradicate the virus from the CNS.

## Neuronal Changes and “Neurodegeneration” in HIVE

In the era of cART, the literature on neuronal death and dropout in HIV-infected people remains highly difficult to judge in terms of its clinical relevancy. The difficulties are quite complex and have been discussed [1]. The essence is that with HIVE, inflammation damages neuronal viability and leads some of the cells to drop out. In patients with HIVE, it can be logically argued that neuronal dropout is contributory to HAND, and that HIV-associated dementia is a type of neurodegenerative disease. Pursuing a “neuroprotective” strategy in model systems that mimic HIVE is of clinical relevance in patients with HIVE. The field continues to hold that, when HIVE is not present, inflammation drives HAND and fuels neurodegeneration. But the same underlying neuropathology in virally suppressed patients without HIVE has never been clearly substantiated [16]. That in turn begs the question of why protecting against neurodegeneration (in parallel with reducing inflammation and viral replication) remains a mainstay in the search for pharmacotherapies for HAND.

If one broadens the definition of neurodegeneration to include nonlethal and/or reversible changes in neuronal function, then neuroprotection of virally suppressed individuals remains a logical therapeutic approach. Evidence which supports this concept includes brain images showing functional adaptation occurring in HIV-infected patients who do not have neurocognitive impairment [17, 18, 19•]. Neurochemical data from autopsy specimens also reveal that nonlethal synaptic accommodation of neurotransmitter systems is related to HAND, including heightened neocortical dopaminergic tone [20•]. Adopting a broader functional definition of neurodegeneration is, however, at odds with the traditional definition of neurodegenerative diseases such as Alzheimer’s or Parkinson’s diseases. Those diseases are defined classically as being due to an irreversible neuronal death, cell dropout, and clear-cut morphological destruction of neuronal elements; their clinical manifestations only occur after a very large proportion of the neuronal elements have been destroyed. Those kinds of neuropathological changes have not been observed in recent times with consistency in patients with HAND, whereas nonlethal changes in neuronal function probably do occur [20•, 21••]. These milder types of changes are likely to underlie the milder neurocognitive impairments that are observed. Abnormal neuronal function (versus death and dropout) is akin to the metabolic types of encephalopathies, which are traditionally described in a completely different chapter of our neuropathology textbooks. Thus, neuroprotection from metabolic types of damage in patients with viral suppression seems an apt description of what is probably happening (versus neuronal death and dropout).

With survival to older ages in virally suppressed patients, roles of biological and pathological brain aging (such as Alzheimer’s or Parkinson’s diseases) have been suggested to be potentially interactive with HAND at the histomorphological level. The underlying hypothesis is that the intersection of age-associated and HIV-associated neurodegeneration could mutually exacerbate each other. The neuropathological literature on this is fragmented, controversial, and inconclusive [22•]. As noted, the case for neuronal death, dropout, and classical neurodegeneration in virally suppressed patients with HAND is increasingly difficult to substantiate. That makes potential synergy with age-related neurodegenerative diseases at the mechanistic level difficult to accept. It is true that neuropathological elements of classical neurodegenerative diseases have begun to appear in older people with HIV infection. And surveys show that elderly people with HIV infection are more vulnerable to neurocognitive impairment. But such changes also begin to occur in aging populations that are not HIV infected. One can just as logically hypothesize that age- and HIV-associated effects could run in parallel with no mechanistic overlapping, and contribute additively to neurocognitive declines in more elderly HIV-infected people. The postulated evolution of a neuropathologically distinct subtype of HAND in people who have age-associated neuropathologies remains to be convincingly validated. It remains unlikely to be disproved completely in the near future, in part because proving a negative proposition is difficult to do and also because negative data can be seen as unwelcome news.

## White Matter Changes in HIVE

White matter damage adds to the spectrum of changes in HIVE. The white matter changes can vary in type, intensity, and their distribution in the brain. White matter edema leads to loss of staining of the myelin (“pallor”). Outright acute destruction of myelin due to exuberant HIV-1 replication and inflammation can occur rarely in HIVE, but more typically slow myelin loss and its phagocytosis by macrophages are restricted to perivascular sectors [23, 24]. Brain imaging studies suggest that focal white matter anomalies are relatively common in HIV-infected patients, including many virally suppressed patients who do not have HIVE, but the neuropsychological implications of that still have not been elucidated clearly [25].

## The Neurovascular Unit and Its Potential Importance in the Cliniconeuropathological Correlation

Gaps in our understanding of the cliniconeuropathological correlation of HAND persist. Each of the neuropathological features reviewed above in the context of their clinical relevancy (or lack of) has been discussed recently [1]. Recent experience has shown convincingly that the overwhelming majority of patients today who are virally suppressed do not have HIVE or MGNE. As well, most of these patients do not have substantial HIV replication in brain tissue [7••] and they do not have an inflammatory gene expression profile in brain tissue [21••]. Of all the CNS structures that are abnormal in virally suppressed patients with HAND, the neurovascular unit (NVU) may be the most important regarding the cliniconeuropathological correlation. The NVU refers to the blood vessel compartment in the CNS and its interface with a web of physical and functional interactions with brain parenchyma [26–28]. The interconnected parts include astrocyte foot processes, neuronal processes and synapses, endothelial cells, pericytes, perivascular macrophages and microglial cells, CNS extracellular spaces, the subarachnoid spaces and cerebrospinal fluid, and perhaps most importantly, blood plasma. Subtle changes in blood plasma are prevalent in HIV-infected patients [29–31], and systemic changes as reflected in the vascular compartment are indeed significantly correlated with HAND. The anemia of chronic inflammation (low red cell volume), and increased plasma lipopolysaccharide due translocation of gut bacteria are both significantly correlated with HAND [32•, 33]. As noted above, the neurovirological correlation in virally suppressed patients with HAND may be weaker than the correlation with plasma viral replication [7••].

The NVU is, therefore, situated anatomically at the critical junction between CNS parenchyma and the plasma compartment at the luminal surface of vascular endothelial cells. Its best-known function is maintaining a highly selective physical barrier between blood plasma and brain parenchyma known as the blood-brain barrier (BBB). In HIVE, the BBB is leaky and leads to cerebral edema by permitting blood plasma to enter the CNS. Experimental systems suggest that this could be detrimental to brain function. Cerebral edema and opening of the BBB is, however, a nonspecific response to CNS injury. It is not clear whether it reflects an epiphenomenon of brain inflammation versus a substantial cause of brain dysfunction in HIVE. As well, we do not know whether it occurs substantially in HAND patients taking cART who do not have HIVE, encephalitis, or other ongoing neuropathological process. Because the BBB is damaged in HIVE, which in turn is correlated with HAND, it is not surprising that the BBB disturbance was correlated with HAND in studies before the era of cART [23].

Beyond acting as a physical barrier, other functions of the NVU could be perturbed in cART-treated patients who are virally suppressed. As noted, endothelial cell surfaces in contact with blood plasma transmit signals to neurons and glia via the NVU (without apparent breakdown in the BBB). These processes may still be perturbed in virally suppressed patients who have low level inflammatory changes in blood plasma [28–31, 32•, 33–35]. Another key role of the NVU is the regulation of microvascular blood flow. The inhibitory neurotransmitter gamma aminobutyric acid (GABA) controls changes in regional microvascular blood flow via the NVU through influences on contractility of pericytes around capillaries and arteriolar smooth muscle [26–28]. Studies using blood oxygenation level-dependent (BOLD) functional magnetic resonance imaging (fMRI) show convincingly that blood flow in specific brain regions is perturbed in cART-treated patients, with and without HAND [19•]. GABAergic transmission is well-known to be abnormally low in HIV-infected brain tissue [21••]. Thus, abnormal GABAergic neural transmission might be involved with the changes in microvascular blood flow in virally suppressed patients.

Compelling evidence that the NVU is probably important in HAND without HIVE was obtained from a recently reported brain gene expression profile [21••]. The study examined over 56,000 gene transcripts in neuropsychologically impaired people with HIVE who lacked CNS viral suppression, and also in people who were virally suppressed in the blood and brain and did not have HIVE. Three different sectors of the brain were examined. Over 1000 brain transcripts were regulated in HAND with HIVE. In sharp contrast, brain gene regulation was highly restricted in virally suppressed patients with HAND without HIVE. These individuals had abnormal transcription regulation limited to a network of brain genes expressed by endothelial cells. Many of these endothelial-type molecules are shed; their concentrations are increased in blood plasma and are not completely restored to normal in virally suppressed patients [35]. The implication overall is that perturbation of neurovascular biology may be a keystone process in patients who are virally suppressed, whereas in the patients without viral suppression, brain inflammation is a more prevalent force. Heightened awareness that there are two types of HAND with divergent neurovirological [7••] and brain transcriptional [21••] patterns was suggested two decades ago before the introduction of effective viral suppression [36].

## Neuropathology and Eradicating Latent HIV in the CNS

In 2015, the HIV/AIDS research enterprise worldwide is engaged in an effort to cure AIDS. The effort is focused on eradicating from the body a small pool of genomically integrated but transcriptionally silent cellular HIV DNA. This “latent” pool of HIV DNA is apparently not diminished using current cART formulations; when cART is stopped, the latent HIV pool “reseeds” the pool of replicating virus in the body [37]. The nature of the latent pool of HIV DNA in the CNS has not been characterized as yet, and some have questioned whether its eradication from the body is scientifically feasible. From neurovirological data obtained at autopsy, it is well documented that genomic HIV DNA is present in many brain specimens. In many patients, there is substantial divergence between the size of the HIV DNA pool and the replicating pool (HIV RNA) [7••]. It is evident that the CNS compartment can be variously enriched with, or depleted of, HIV DNA relative to the viral replication rate. Since cells that host latent HIV are likely to be immunologically “inert” and invulnerable to immune surveillance [37], there is very little reason to believe that pathological inflammation such as that observed in HIVE would be linked to it. Similarly, “neurodegeneration” driven by CNS inflammation would be unlikely to be associated with the CNS pool of latent HIV DNA. A recent report suggested that a transcriptionally silenced HIV DNA pool in 10 infected brain specimens was related to high expression of the microglial cell transcription factor BCL11b [38]. It awaits independent replication in more patients, yet intriguingly suggests that repressing the pool of latent HIV DNA in microglial cells is potentially feasible.

Whether HAND is associated with enrichment of the CNS pool of latent HIV DNA (relative to the CNS replication rate) is not clear. Current thinking on this topic is speculative at best. Available data on HIV RNA and total HIV DNA from autopsy brain specimens show that a very low viral replication rate (and low total HIV DNA concentration) in the CNS, as seen in virally suppressed patients, is not substantially correlated with HAND. In contrast, very high rates of viral replication in the CNS (with high concentrations of HIV DNA), as seen in HIVE patients not taking cART, are correlated significantly with HAND [7••]. Inclusion of the latter type of patients in most surveys is what drives the significance of this correlation overall. In a follow-up to that study, the authors measured integrated HIV DNA (a more specific measure of viral latency in tissue) using the two-step Alu-gag assay in 29 of the brain specimens. The neurovirological correlation using integrated HIV DNA (both the coefficient and the regression line) was not different from results using total HIV DNA or HIV RNA. The result favors the hypothesis that the latent pool of HIV in the brain per se is no more influential to the cliniconeurovirological correlation than are the other two viral pools that were measured (the author’s unpublished observation).

## Conclusions: the Future

The neuropathology of HIV infection changed drastically when cART was introduced into clinical practice. The neuroAIDS field clings to most tenets of the “old” pathology of unsuppressed patients, especially the concepts that encephalitis, neurodegeneration, and CNS HIV replication are the key drivers of HAND. Once considered seminal to the field, those tenets are of increasingly lower relevance clinically. The lack of specific pathoanatomical changes in virally suppressed patients means that the cliniconeuropathological correlation of HAND is driven by subtler and potentially reversible neurometabolic changes, such as those associated with synaptic transmission and neural plasticity [20•]. Correlation between HAND and systemic pathologies also implies a possible metabolic encephalopathy that is one component of a systemically driven illness. Establishing a new conceptual framework is painful and risks “throwing the baby out with the bath water.” But the NeuroAIDS field is ripe for a change. The next generation of practitioners and scientists faces a very stiff headwind to meet that challenge.
